# Bis(2-hydroxy­imino­methyl-6-methoxy­phenolato-κ^2^ *O* ^1^,*N*)cobalt(II)

**DOI:** 10.1107/S1600536808039512

**Published:** 2008-11-29

**Authors:** Shu Hua Zhang, Cheng Min Ge, Chao Feng

**Affiliations:** a Key Laboratory of Non-Ferrous Metal Materials and Processing Technology, Department of Materials and Chemical Engineering, Guilin University of Technology, Ministry of Education, Guilin 541004, People’s Republic of China

## Abstract

In the title compound, [Co(C_8_H_8_NO_3_)_2_], the Co^II^ atom lies on a centre of inversion and is coordinated in a slightly distorted square-planar geometry by two N and two O atoms from the 2-hydroxy­imino­methyl-6-methoxy­phenolate ligands. Intra­molecular O—H⋯O hydrogen bonds are formed and the complexes form stacks along the *b* axis, with an inter­planar separation of 3.332 (1) Å between complexes. Pairs of C—H⋯O contacts are formed between complexes in neighbouring stacks.

## Related literature

For recent related literature concerning Schiff-base compounds, see: Gupta & Sutar (2008 ▶); Sreenivasulu *et al.* (2005 ▶); Zhang *et al.* (2008 ▶); Raptopoulou *et al.* (2006 ▶); Milios *et al.* (2006 ▶); Yang *et al.* (2007 ▶).
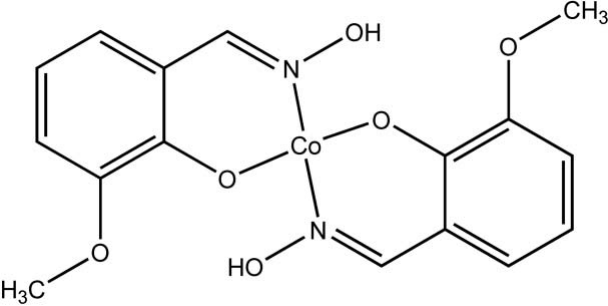


## Experimental

### 

#### Crystal data

[Co(C_8_H_8_NO_3_)_2_]
*M* *_r_* = 391.24
Monoclinic, 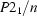

*a* = 8.4254 (19) Å
*b* = 4.9111 (11) Å
*c* = 18.951 (4) Å
β = 95.375 (3)°
*V* = 780.7 (3) Å^3^
*Z* = 2
Mo *K*α radiation
μ = 1.14 mm^−1^
*T* = 293 (2) K
0.22 × 0.18 × 0.14 mm

#### Data collection

Bruker SMART CCD diffractometer
Absorption correction: none
4577 measured reflections
1433 independent reflections
1216 reflections with *I* > 2σ(*I*)
*R*_int_ = 0.021

#### Refinement

*R*[*F* ^2^ > 2σ(*F* ^2^)] = 0.025
*wR*(*F* ^2^) = 0.065
*S* = 1.04
1433 reflections
117 parameters
H-atom parameters constrained
Δρ_max_ = 0.23 e Å^−3^
Δρ_min_ = −0.17 e Å^−3^

### 

Data collection: *SMART* (Bruker, 2000 ▶); cell refinement: *SAINT* (Bruker, 2000 ▶); data reduction: *SAINT*; program(s) used to solve structure: *SHELXS97* (Sheldrick, 2008 ▶); program(s) used to refine structure: *SHELXL97* (Sheldrick, 2008 ▶); molecular graphics: *SHELXTL* (Sheldrick, 2008 ▶); software used to prepare material for publication: *SHELXTL*.

## Supplementary Material

Crystal structure: contains datablocks global, I. DOI: 10.1107/S1600536808039512/bi2324sup1.cif

Structure factors: contains datablocks I. DOI: 10.1107/S1600536808039512/bi2324Isup2.hkl

Additional supplementary materials:  crystallographic information; 3D view; checkCIF report

## Figures and Tables

**Table 1:** Hydrogen-bond geometry (Å, °)

*D*—H⋯*A*	*D*—H	H⋯*A*	*D*⋯*A*	*D*—H⋯*A*
O2—H2⋯O1^i^	0.82	1.91	2.5336 (19)	132
C7—H7⋯O2^ii^	0.93	2.48	3.321 (2)	150

Symmetry codes: (i) 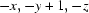; (ii) 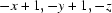.

**Fig. 1.**
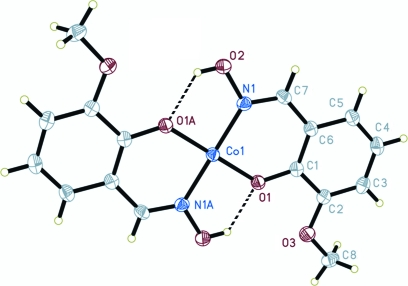
Molecular structure of the title compound, showing 30% probability displacement ellipsoids for non-H atoms. Dashed lines denote O—H···O hydrogen bonds. Symmetry code (A): -x, 1 - y, -z.

**Fig. 2.**
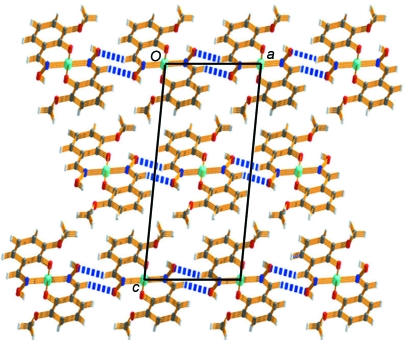
Packing diagram viewed down the b axis. Dasehd lines denote C—H···O contacts.

## supplementary crystallographic information

### Comment

Schiff-base complexes have been studied for many years (Gupta & Sutar, 2008; Sreenivasulu *et al.*, 2005; Zhang *et al.*, 2008) and have aroused increasing interest because of their antiviral, anticancer, catalytic and fluorescent properties. Most model studies of metal complexes of Schiff-base ligands containing salicylaldehyde derivatives and oxime have focused on the binding mode of the ligands (Raptopoulou *et al.*, 2006; Milios *et al.*, 2006; Yang *et al.*, 2007). The crystal structures of the complexes demonstrate that the Schiff-base ligands act in a bidentate, tridentate or mu^5^:eta^1^:etaî^:eta^3^ mode, coordinating through the phenolato O, imine N, or oxime O atoms. Our research group is interested in the Schiff-base derived from 2-hydroxy-3-methoxy-benzaldehyde and hydroxylammonium chloride.

### Experimental

A solution of (0.152 g, 1.0 mmol) 2-hydroxy-3-methoxy-benzaldehyde oxime and (0.056 g, 1 mmol) potassium hydroxide in 20 ml absolute methanol was added slowly to a solution of CoNO_3_.6H_2_O (0.145 g, 0.5 mmol) in methanol. The mixture was stirred for 1 h at room temperature to give a red solution which was filtered and the filtrate was left to stand at room temperature. Red block crystals suitable for were obtained by slow evaporation Yield: 80.1 % (based on Co). Elemental analysis calculated: C 49.12, H 4.12, N 7.16 %; found: C 48.99, H 4.21, N 7.22 %.

### Refinement

H atoms were positioned geometrically and refined with a riding model, with distances 0.96 (CH_3_) or 0.93 Å (aromatic ring), and with *U*_iso_(H) = 1.2 *U*_eq_(aromatic ring) or *U*_iso_(H) = 1.5 *U*_eq_(CH_3_), and with O–H distance 0.82 Å and *U*_iso_(H) = 1.5 *U*_eq_(O).

### Crystal data

**Table:**

[Co(C_8_H_8_NO_3_)_2_]	*F*_000_ = 402
*M**_r_* = 391.24	*D*_x_ = 1.664 Mg m^−^^3^
Monoclinic, *P*2_1_/*n*	Mo *K*α radiation λ = 0.71073 Å
Hall symbol: -P 2yn	Cell parameters from 4577 reflections
*a* = 8.4254 (19) Å	θ = 2.6–25.5º
*b* = 4.9111 (11) Å	µ = 1.14 mm^−^^1^
*c* = 18.951 (4) Å	*T* = 293 (2) K
β = 95.375 (3)º	Block, red
*V* = 780.7 (3) Å^3^	0.22 × 0.18 × 0.14 mm
*Z* = 2	

### Data collection

**Table:**

Bruker SMART CCD diffractometer	1216 reflections with *I* > 2σ(*I*)
Radiation source: fine-focus sealed tube	*R*_int_ = 0.021
Monochromator: graphite	θ_max_ = 25.5º
*T* = 293(2) K	θ_min_ = 2.6º
φ and ω scans	*h* = −10→10
Absorption correction: none	*k* = −5→5
4577 measured reflections	*l* = −22→22
1433 independent reflections	

### Refinement

**Table:**

Refinement on *F*^2^	Secondary atom site location: difference Fourier map
Least-squares matrix: full	Hydrogen site location: inferred from neighbouring sites
*R*[*F*^2^ > 2σ(*F*^2^)] = 0.025	H-atom parameters constrained
*wR*(*F*^2^) = 0.065	*w* = 1/[σ^2^(*F*_o_^2^) + (0.0303*P*)^2^ + 0.2885*P*] where *P* = (*F*_o_^2^ + 2*F*_c_^2^)/3
*S* = 1.04	(Δ/σ)_max_ < 0.001
1433 reflections	Δρ_max_ = 0.23 e Å^−^^3^
117 parameters	Δρ_min_ = −0.17 e Å^−^^3^
Primary atom site location: structure-invariant direct methods	Extinction correction: none

### Special details

**Table:**

Geometry. All esds (except the esd in the dihedral angle between two l.s. planes) are estimated using the full covariance matrix. The cell esds are taken into account individually in the estimation of esds in distances, angles and torsion angles; correlations between esds in cell parameters are only used when they are defined by crystal symmetry. An approximate (isotropic) treatment of cell esds is used for estimating esds involving l.s. planes.
Refinement. Refinement of F^2^ against ALL reflections. The weighted R-factor wR and goodness of fit S are based on F^2^, conventional R-factors R are based on F, with F set to zero for negative F^2^. The threshold expression of F^2^ > 2sigma(F^2^) is used only for calculating R-factors(gt) etc. and is not relevant to the choice of reflections for refinement. R-factors based on F^2^ are statistically about twice as large as those based on F, and R- factors based on ALL data will be even larger.

### Fractional atomic coordinates and isotropic or equivalent isotropic displacement parameters (Å^2^)

**Table:**

	*x*	*y*	*z*	*U*_iso_*/*U*_eq_	
Co1	0.0000	0.5000	0.0000	0.03262 (15)	
C1	0.1093 (2)	0.0793 (4)	0.09770 (10)	0.0353 (4)	
C2	0.0701 (2)	−0.1107 (4)	0.14960 (10)	0.0377 (5)	
C3	0.1851 (3)	−0.2781 (4)	0.18262 (11)	0.0448 (5)	
H3	0.1581	−0.4028	0.2164	0.054*	
C4	0.3423 (3)	−0.2610 (4)	0.16538 (11)	0.0477 (6)	
H4	0.4196	−0.3744	0.1880	0.057*	
C5	0.3837 (3)	−0.0794 (4)	0.11564 (11)	0.0429 (5)	
H5	0.4889	−0.0696	0.1048	0.052*	
C6	0.2676 (2)	0.0939 (4)	0.08055 (10)	0.0361 (4)	
C7	0.3153 (2)	0.2796 (4)	0.02809 (10)	0.0386 (5)	
H7	0.4217	0.2799	0.0187	0.046*	
C8	−0.1391 (3)	−0.3153 (5)	0.20761 (12)	0.0535 (6)	
H8A	−0.1090	−0.4897	0.1902	0.080*	
H8B	−0.2529	−0.3074	0.2077	0.080*	
H8C	−0.0903	−0.2898	0.2550	0.080*	
N1	0.21926 (19)	0.4465 (3)	−0.00691 (8)	0.0363 (4)	
O1	−0.00708 (15)	0.2354 (3)	0.06754 (7)	0.0387 (3)	
O2	0.29495 (16)	0.6086 (3)	−0.05443 (8)	0.0487 (4)	
H2	0.2301	0.7143	−0.0744	0.073*	
O3	−0.08737 (17)	−0.1063 (3)	0.16291 (8)	0.0494 (4)	

### Atomic displacement parameters (Å^2^)

**Table:**

	*U*^11^	*U*^22^	*U*^33^	*U*^12^	*U*^13^	*U*^23^
Co1	0.0291 (2)	0.0337 (2)	0.0348 (2)	−0.00160 (15)	0.00146 (15)	0.00203 (16)
C1	0.0385 (11)	0.0311 (10)	0.0354 (11)	0.0001 (8)	−0.0005 (9)	−0.0038 (8)
C2	0.0415 (11)	0.0352 (10)	0.0362 (11)	−0.0016 (9)	0.0025 (9)	−0.0015 (9)
C3	0.0566 (14)	0.0375 (11)	0.0392 (11)	−0.0009 (10)	−0.0007 (10)	0.0040 (9)
C4	0.0503 (13)	0.0425 (12)	0.0478 (13)	0.0103 (10)	−0.0080 (10)	−0.0001 (10)
C5	0.0384 (11)	0.0455 (12)	0.0436 (12)	0.0057 (9)	−0.0030 (9)	−0.0045 (10)
C6	0.0360 (10)	0.0350 (10)	0.0364 (11)	−0.0010 (8)	−0.0017 (8)	−0.0043 (8)
C7	0.0286 (10)	0.0438 (11)	0.0429 (11)	0.0001 (9)	0.0016 (8)	−0.0041 (9)
C8	0.0591 (14)	0.0510 (14)	0.0524 (13)	−0.0079 (11)	0.0160 (11)	0.0078 (11)
N1	0.0327 (9)	0.0410 (10)	0.0352 (9)	−0.0053 (7)	0.0040 (7)	0.0019 (7)
O1	0.0332 (7)	0.0397 (8)	0.0434 (8)	0.0005 (6)	0.0044 (6)	0.0083 (6)
O2	0.0346 (8)	0.0598 (10)	0.0523 (9)	−0.0032 (7)	0.0070 (7)	0.0185 (8)
O3	0.0452 (9)	0.0493 (8)	0.0547 (9)	−0.0010 (7)	0.0105 (7)	0.0153 (8)

### Geometric parameters (Å, °)

**Table:**

Co1—O1	1.8290 (13)	C4—H4	0.930
Co1—O1^i^	1.8290 (13)	C5—C6	1.414 (3)
Co1—N1	1.8826 (17)	C5—H5	0.930
Co1—N1^i^	1.8826 (17)	C6—C7	1.434 (3)
C1—O1	1.331 (2)	C7—N1	1.290 (2)
C1—C6	1.404 (3)	C7—H7	0.930
C1—C2	1.417 (3)	C8—O3	1.425 (2)
C2—O3	1.374 (2)	C8—H8A	0.960
C2—C3	1.376 (3)	C8—H8B	0.960
C3—C4	1.396 (3)	C8—H8C	0.960
C3—H3	0.930	N1—O2	1.400 (2)
C4—C5	1.367 (3)	O2—H2	0.820
			
O1—Co1—O1^i^	180.00 (8)	C6—C5—H5	119.8
O1—Co1—N1	92.64 (6)	C1—C6—C5	119.37 (19)
O1^i^—Co1—N1	87.36 (6)	C1—C6—C7	121.78 (18)
O1—Co1—N1^i^	87.36 (6)	C5—C6—C7	118.86 (18)
O1^i^—Co1—N1^i^	92.64 (6)	N1—C7—C6	123.89 (18)
N1—Co1—N1^i^	180.00 (13)	N1—C7—H7	118.1
O1—C1—C6	123.29 (18)	C6—C7—H7	118.1
O1—C1—C2	117.87 (18)	O3—C8—H8A	109.5
C6—C1—C2	118.84 (17)	O3—C8—H8B	109.5
O3—C2—C3	125.24 (19)	H8A—C8—H8B	109.5
O3—C2—C1	114.13 (17)	O3—C8—H8C	109.5
C3—C2—C1	120.62 (19)	H8A—C8—H8C	109.5
C2—C3—C4	120.0 (2)	H8B—C8—H8C	109.5
C2—C3—H3	120.0	C7—N1—O2	112.97 (16)
C4—C3—H3	120.0	C7—N1—Co1	128.68 (14)
C5—C4—C3	120.65 (19)	O2—N1—Co1	118.33 (12)
C5—C4—H4	119.7	C1—O1—Co1	129.71 (13)
C3—C4—H4	119.7	N1—O2—H2	109.5
C4—C5—C6	120.5 (2)	C2—O3—C8	116.90 (17)
C4—C5—H5	119.8		

Symmetry codes: (i) −*x*, −*y*+1, −*z*.

### Hydrogen-bond geometry (Å, °)

**Table:**

*D*—H···*A*	*D*—H	H···*A*	*D*···*A*	*D*—H···*A*
O2—H2···O1^i^	0.82	1.91	2.5336 (19)	132
C7—H7···O2^ii^	0.93	2.48	3.321 (2)	150

Symmetry codes: (i) −*x*, −*y*+1, −*z*; (ii) −*x*+1, −*y*+1, −*z*.
